# Supplementary material for: Impact of an Agriphotovoltaic System on Metabolites and the Sensorial Quality of Cabbage (Brassica oleracea var. capitata) and Its High-Temperature-Extracted Juice
Source: Foods. 2022 Feb 9;11(4):498. doi: 10.3390/foods11040498 (PMC8870755; doi:10.3390/foods11040498)
Supplement: Supplementary file 1 [file foods-11-00498-s001.zip › foods-1575661-supplementary-done.pdf]

foods

Impact of an Agriphotovoltaic System on Metabolites and the Sensorial Quality of Cabbage (*Brassica oleracea* var. *capitata*) and Its High-Temperature-Extracted Juice

Hyeon-Woo Moon[1,2] and Kang-Mo Ku[1,2,*]

[1] Department of Horticulture at Chonnam National University, Gwangju 61186, Republic of Korea
[2] BK21 Interdisciplinary Program in IT-Bio Convergence System, Chonnam National University, Gwangju 61186, Republic of Korea; notorious931216@gmail.com (H.W.M.)
* Correspondence: ku9@jnu.ac.kr (K.M.K.)

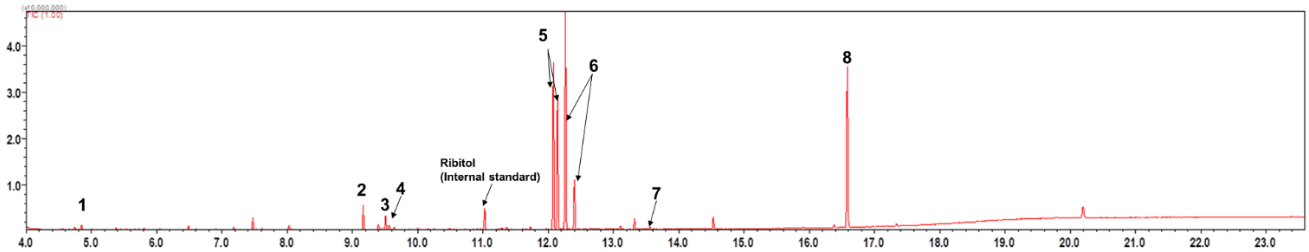

Figure S1. Chromatogram of water-soluble primary metabolites. The numbers above the peaks correspond to those mentioned in Table S1. Metabolites were identified to have more than 80% similarity in the NIST library.

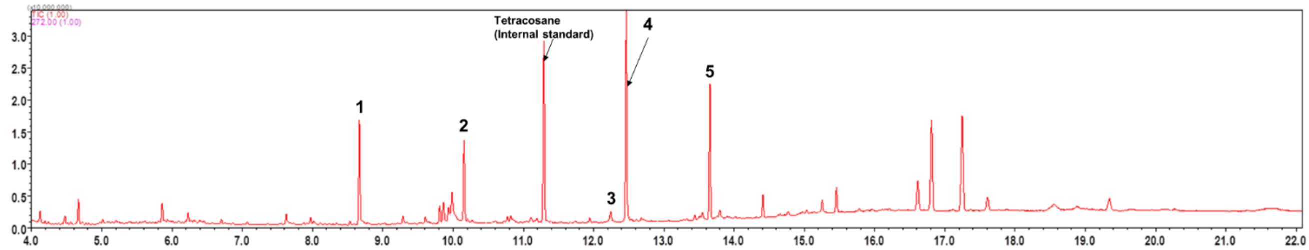

Figure S2. Chromatogram of lipid-soluble primary metabolites. The numbers above the peaks correspond to those mentioned in Table S2. Metabolites were identified to have more than 80% similarity in the NIST library.

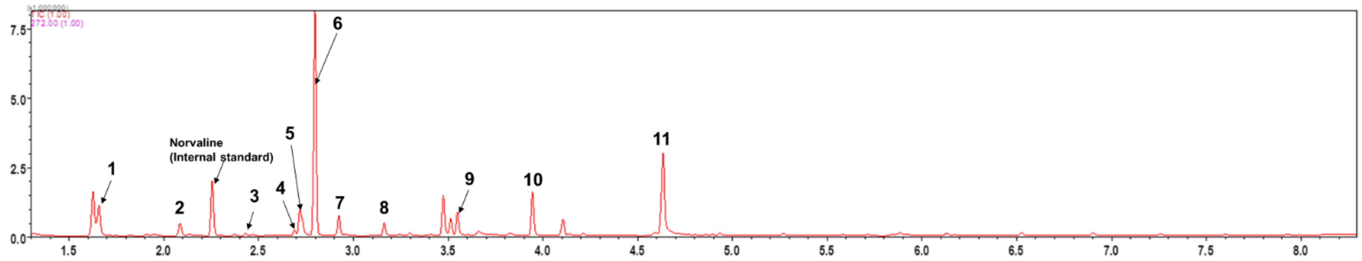

Figure S3. Chromatogram of amino acids. The numbers above the peaks correspond to those mentioned in Table S3. Metabolites were identified to have more than 80% similarity in the NIST library.

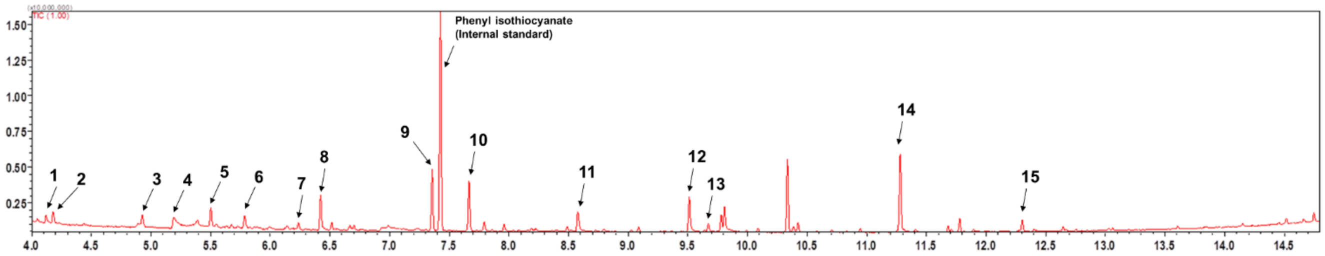

Figure S4. Chromatogram of glucosinolate hydrolysis products of cabbage juice. The numbers above the peaks correspond to those mentioned in Table S4.

Table S1. Identification of water-soluble compounds in cabbage.

No.	Compound	RT (min)	Characteristic ion (*m*/*z*)	Derivatization[a]	Identification[c]
1	1,3-Propanediol	4.85	45, 73, 115, 130, **147**[b]	2TMS	NIST
2	Malic acid	9.17	45, 73, **147**	3TMS	NIST
3	L-5-oxoproline	9.51	45, 73, 147, **156**	2TMS	NIST
4	4-Aminobutanoic acid	9.56	45, 73, 147, **174**, 304	3TMS	NIST
5	Fructose	12.08	73, 103, 147, **217**, 307	5TMS, Meox	NIST
6	Glucose	12.26	**73**, 147, 160, 205, 319	5TMS, Meox	NIST
7	Myo-inositol	13.56	73, 147, 191, 217, 305, 318	6TMS	NIST
8	Sucrose	16.58	73, 103, 147, 191, 217, **361**	8TMS	NIST

[a] TMS, trimethylsilyl; Meox, methyloxime
[b] Bold is the label of highest peak
[c] Metabolites were identified using standard compound (STD).

Table S2. Identification of lipid-soluble compounds in cabbage.

No.	Compound	RT (min)	Characteristic ion (*m*/*z*)	Derivatization[a]	Identification[c]
1	Palmitic acid	8.67	**73**[b], 75, 117, 129, 132, 145	TMS	NIST
2	Stearic acid	10.16	73, 75, 117, 132, 145, **341**	TMS	NIST
3	2-Palmitoylglycerol	12.25	43, 57, 73, 103, **129**, 147, 191, 218, 239, 313	2TMS	NIST
4	1-Monopalmitin	11.30	43, 57, 73, 129, 147, 203, 239, **371**	2TMS	NIST
5	Glycerol monostearate	13.66	43, 57, 73, 129, 147, 203, 267, **399**	2TMS	NIST

[a] TMS, trimethylsilyl. [b] Bold is the label of base peak. [c] Metabolites were identified using standard compound (STD).

Table S3. Identification of amino acids in cabbage.

No.	Compound	RT (min)	Characteristic ion (*m*/*z*)	Derivatization[a]	Identification[c]
1	Alanine	1.66	70, 88, 116, **130**[b]	PCF	NIST, STD
2	Valine	2.08	55, 72, 98, **116**, 158	PCF	NIST, STD
3	Isoleucine	2.43	57, 74, 86, 101, 130, **172**	PCF	NIST, STD
4	Threonine	2.69	56, 74, **101**, 143, 160, 203	PCF	NIST, STD
5	3-Aminobutyric acid	2.72	63, 88, 112, 130, 144, **172**, 216	PCF	NIST, STD
6	Proline	2.80	70, 114, **156**	PCF	NIST, STD
7	Asparagine	2.92	69, 113, 141, **155**	PCF	STD
8	S-methyl-L-cysteine	3.16	61, 74, 118, **160**, 176	PCF	NIST
9	Aspartic acid	3.55	70, 88, 114, 130, 156, **216**	PCF	NIST, STD
10	Glutamic acid	3.95	56, 84, 100, 142, 155, **170**, 230	PCF	NIST, STD
11	Glutamine	4.63	56, 59, **84**, 142, 187	PCF	NIST, STD

[a] PCF, propyl chloroformate

[b] Bold is the label of base peak

[c] Metabolites were identified using standard compound (STD) and the library of the National Institute of Standards and Technology (NIST).

Table S4. Identification of glucosinolate hydrolysis products and organic volatile compounds in cabbage.

No.	Compound	RT (min)	Characteristic ion (*m*/*z*)	Identification[b]
1	2-Methyl pyrazine	4.11	40, 67, **94**[a]	NIST, STD
2	Furfural	4.17	67, 95, **96**	NIST, STD
3	2,5-Dimethyl pyrazine	4.92	**42**, 81, 108	NIST, STD
4	Crembene	5.18	41, **57**	NIST,[1]
5	Dimethyl trisulfide	5.49	45, 79, 111, **126**	NIST, STD
6	1H-pyrrole-2-carboxaldehyde	5.78	40, 66, **95**	NIST, STD
7	S-methyl methanthiosulfonate	6.23	**47**, 63, 81, 94, 109, 126	NIST, STD
8	Iberverin nitrile	6.41	41, **61**, 115	NIST,[2]
9	Erucin nitrile	7.35	**61**, 82, 129	NIST,[3]
10	3-Phenylpropanenitrile	7.66	51, 65, **91**, 131	NIST
11	Iberin nitrile	8.57	**41**, 64, 131	NIST,[3]
12	Sulforaphane nitrile	9.50	41, **55**, 64, 82, 145	NIST,[2]
13	Goitrin	9.66	41, 68, **129**	NIST,[1,3]
14	Indole-3-acetonitrile	11.27	51, 77, 101, 130, **155**	NIST,[2]
15	4-Methoxyindole-3-acetonitrile	12.30	63, 89, 116, 143, **171**, 186	NIST

[a] Bold is the label of base peak

[b] Metabolites were identified using standard compound (STD), the library of the National Institute of Standards and Technology (NIST), and in comparison with mass spectra (MS) in the literature.

Table S5. The amount of primary metabolites in cabbage and juice.

		Fresh (μg·g⁻¹ DW)		Juice (μg·mL⁻¹)	
Sort	Compound	OF	APV	OF	APV
Water-soluble[a]	1,3-Propanediol	3.55 ± 0.4	3.56 ± 0.2	0.93 ± 0.0	0.90 ± 0.1
Water-soluble	Malic acid	17.29 ± 1.1	18.26 ± 1.8	3.92 ± 0.1	4.47 ± 0.0
Water-soluble	5-Oxoproline	10.96 ± 1.2	10.34 ± 0.4	3.46 ± 0.1	3.34 ± 0.1
Water-soluble	4-Aminobutanoic acid	3.95 ± 0.5	5.31 ± 1.0	1.12 ± 0.0	1.19 ± 0.0
Water-soluble	Fructose	240.9 ± 1.8	231.1 ± 25.1	30.44 ± 0.9	27.62 ± 0.9
Water-soluble	Glucose	224.5 ± 4.8	216.0 ± 20.1	29.49 ± 1.0	26.21 ± 0.6
Water-soluble	Myo-inositol	2.03 ± 0.2	2.27 ± 0.3	0.25 ± 0.0	0.29 ± 0.0
Water-soluble	Sucrose	135.0 ± 16.8	169.2 ± 33.9	11.73 ± 0.7	15.28 ± 1.0
Lipid-soluble	Palmitic Acid	0.73 ± 0.2	0.92 ± 0.1	0.36 ± 0.1	0.24 ± 0.0
Lipid-soluble	Stearic acid	0.61 ± 0.1	0.74 ± 0.1	0.30 ± 0.0	0.21 ± 0.0
Lipid-soluble	2-Palmitoylglycerol	0.14 ± 0.0	0.17 ± 0.0	0.07 ± 0.0	0.05 ± 0.0
Lipid-soluble	1-Monopalmitin	1.70 ± 0.5	2.37 ± 0.4	0.77 ± 0.1	0.61 ± 0.0
Lipid-soluble	Glycerol monostearate	1.10 ± 0.3	1.51 ± 0.2	0.48 ± 0.0	0.39 ± 0.0
Amino acid	Alanine	1.03 ± 0.3	1.12 ± 0.1	0.94 ± 0.1	1.12 ± 0.0
Amino acid	Valine	0.49 ± 0.1	0.57 ± 0.1	0.59 ± 0.0	0.73 ± 0.0
Amino acid	Isoleucine	0.14 ± 0.1	0.11 ± 0.0	0.15 ± 0.0	0.13 ± 0.0
Amino acid	Threonine	0.16 ± 0.0	0.15 ± 0.0	0.17 ± 0.0	0.19 ± 0.0
Amino acid	3-Aminobutyric acid	0.99 ± 0.3	1.09 ± 0.2	1.70 ± 0.2	1.88 ± 0.1
Amino acid	Proline	7.24 ± 0.5	6.17 ± 0.3	7.53 ± 0.9	7.16 ± 0.2
Amino acid	Asparagine	0.63 ± 0.1	0.62 ± 0.1	0.63 ± 0.1	0.66 ± 0.0
Amino acid	S-Methyl-L-cysteine	0.47 ± 0.1	0.44 ± 0.1	0.23 ± 0.0	0.30 ± 0.0
Amino acid	Aspartic acid	0.89 ± 0.0	0.76 ± 0.0	0.74 ± 0.1	0.69 ± 0.0
Amino acid	Glutamic acid	1.66 ± 0.3	1.38 ± 0.1	0.58 ± 0.1	0.61 ± 0.0
Amino acid	Glutamine	3.24 ± 0.6	3.11 ± 0.8	0.23 ± 0.1	0.17 ± 0.0

[a] Water soluble, lipid soluble, and amino acid were quantified by equivalent of ribitol, tetracosane, and norvaline respectively.

References

1. Kim, M.J.; Chiu, Y.-C.; Kim, N.K.; Park, H.M.; Lee, C.H.; Juvik, J.A.; Ku, K.-M. Cultivar-specific changes in primary and secondary metabolites in pak choi (Brassica rapa, Chinensis group) by methyl jasmonate. *International journal of molecular sciences* **2017**, *18*, 1004.
2. Vaughn, S.F.; Berhow, M.A. Glucosinolate hydrolysis products from various plant sources: pH effects, isolation, and purification. *Industrial Crops and Products* **2005**, *21*, 193-202.
3. Oloyede, O.O.; Wagstaff, C.; Methven, L. Influence of cabbage (Brassica oleracea) accession and growing conditions on myrosinase activity, glucosinolates and their hydrolysis products. *Foods* **2021**, *10*, 2903.
